# Double Domain Swapping in Bovine Seminal RNase: Formation of Distinct N- and C-swapped Tetramers and Multimers with Increasing Biological Activities

**DOI:** 10.1371/journal.pone.0046804

**Published:** 2012-10-11

**Authors:** Giovanni Gotte, Alexander Mahmoud Helmy, Carmine Ercole, Roberta Spadaccini, Douglas V. Laurents, Massimo Donadelli, Delia Picone

**Affiliations:** 1 Dipartimento di Scienze della Vita e della Riproduzione, Sezione di Chimica Biologica, Università degli Studi di Verona, Verona, Italy; 2 Dipartimento di Scienze Chimiche, Università degli Studi di Napoli “Federico II”, Naples, Italy; 3 Dipartimento di Scienze Biologiche e Ambientali, Università del Sannio, Benevento, Italy; 4 Instituto de Química Física “Rocasolano” (C.S.I.C.), Madrid, Spain; Universitat Autònoma de Barcelona, Spain

## Abstract

Bovine seminal (BS) RNase, the unique natively dimeric member of the RNase super-family, represents a special case not only for its additional biological actions but also for the singular features of 3D domain swapping. The native enzyme is indeed a mixture of two isoforms: M = M, a dimer held together by two inter-subunit disulfide bonds, and MxM, 70% of the total, which, besides the two mentioned disulfides, is additionally stabilized by the swapping of its N-termini.

When lyophilized from 40% acetic acid, BS-RNase oligomerizes as the super-family proto-type RNase A does. In this paper, we induced BS-RNase self-association and analyzed the multimers by size-exclusion chromatography, cross-linking, electrophoresis, mutagenesis, dynamic light scattering, molecular modelling. Finally, we evaluated their enzymatic and cytotoxic activities.

Several BS-RNase domain-swapped oligomers were detected, including two tetramers, one exchanging only the N-termini, the other being either N- or C-swapped. The C-swapping event, confirmed by results on a BS-K113N mutant, has been firstly seen in BS-RNase here, and probably stabilizes also multimers larger than tetramers.

Interestingly, all BS-RNase oligomers are more enzymatically active than the native dimer and, above all, they display a cytotoxic activity that definitely increases with the molecular weight of the multimers. This latter feature, to date unknown for BS-RNase, suggests again that the self-association of RNases strongly modulates their biological and potentially therapeutic properties.

## Introduction

Bovine seminal ribonuclease (BS-RNase) is the sole natively dimeric [1] member of the pancreatic-type RNase super-family [2], and it is a mixture of two isoforms. The first, called M = M, accounts for about the 30% of the total, and it is dimeric due to two anti-parallel disulfide bonds linking the Cys-31 and 32 of the two subunits [3], [4]; the second isoform, called MxM, (70% of the total) is stabilized, in addition to the mentioned disulfides, also by the three dimensional (3D) swapping [5] of its N-terminal domains (residues 1–15) [6]. BS-RNase is endowed with special biological actions, especially a potentially therapeutic antitumor activity [7]. Notably, only MxM is selectively [8] cytotoxic against malignant cells [9], because it maintains the dimeric structure necessary to evade the RNase inhibitor (RI) even under the reducing cytosolic environment that breaks the inter-subunits disulfide bonds and causes the disassociation of the unswapped M = M form [9], [10].

Libonati first showed that, when dissolved in 40–50% acetic acid (HAc) and subjected to lyophilization [11], BS-RNase forms a mixture of meta-stable oligomeric aggregates [12], whose stability increases in sodium phosphate buffers (NaPi), as occurs to bovine pancreatic ribonuclease (RNase A), the monomeric proto-type of the super-family [11]. Later, Mazzarella and colleagues hypothesized the existence of more than one tetrameric isoform (dimer+dimer) ascribable to different induced orientations of the BS-RNase N-termini [13]. Nevertheless, despite many studies focused on the propensity to natively swap its N-termini [14]–[18] no additional investigations on BS-RNase oligomers have been performed. However, the high sequence identity (about 82%) existing between BS-RNase and RNase A [4], and the similar chromatographic behavior of the two proteins after their multimerization [12], [13], has lead us to hypothesize that BS-RNase could oligomerize through the same mechanism of its pancreatic monomeric counterpart, i.e. the double domain swapping [19] of both N- and/or C-termini [20], [21].

It is noteworthy that the 3D domain swapping mechanism is shared by several fibril-forming proteins, such as cystatin C [22], [23], human prion protein [24], [25], T7-Endonuclase I [26], β-2 microglobulin [27], [28], but also by proteins that are not fibrillogenic, such as cytochrome c [29], and RNase A [20]. By the way, RNase A is considered a model for the formation of amyloid or amyloid-like fibrils through domain swapping [19], although only mutants containing poly-Q- or poly-G-expanded loops were shown to produce native-like fibrils [30], [31]. Contrarily, no conditions yielded fibrils from wt RNase A [31], [32], even if this enzyme displays more than one cross-β-spine-prone sequence [33], and although more recently another pancreatic-type [2] RNase, the eosinophil cationic protein (ECP), was shown to form fibrils [34]. Anyway, RNase A can form several N-or C-domain-swapped oligomers [20], [21] if it is lyophilized from 40% HAc solutions [11], or if highly concentrated protein solutions are subjected to a thermally-induced aggregation procedure [35]. In addition, it has recently been discovered that minor but not negligible amounts of dimers, especially the C-swapped one, are produced *in vitro* in CHO cells and *in vivo* in bovine pancreatic tissue [36]. The structures of RNase A N-swapped or C-swapped dimers, or of a C-swapped cyclic trimer, called N_D_, C_D_ and C_T_ respectively [20], have been solved (PDB codes 1A2W, 1F0V, 1JS0, respectively) [37]–[39], while plausible models have been proposed for RNase A tetramers and larger oligomers [21], [40], [41]. Although being meta-stable, all multimers maintain and/or increase their enzymatic and biological activities, becoming also cytotoxic *in vitro* and *in vivo* [42], depending on their mass (i.e. degree of oligomerization), and on the number and/or exposure of basic charges, these latter features differently related to N- or C-swapped structures [20].

In this composite scenario, the characterization of BS-RNase oligomerization profile could further elucidate the structural determinants controlling the self-association of RNase(s) and of proteins in general [43]. In addition, considering the cytotoxic potential of the seminal enzyme [8], it would be very interesting to see if also BS-RNase multimers display augmented catalytic and/or, above all, biological activities. Thus, we induced BS-RNase multimerization through the mentioned protocols [11], [35], and the oligomers produced were purified and studied in their structural and functional properties, side-by-side with the well characterized RNase A oligomers [20], [21], [37]–[40], employed in this study as standards.

## Results and Discussion

### BS-RNase aggregation, purification, and analysis of the oligomeric products

To avoid spontaneous deamidation, heterogeneity of the samples and side-reactions, the protein used here as wt is N67D-BS-RNase [44] (See Materials and Methods), considering that the biological and structural features of this protein are known to be almost identical to those of the native enzyme [45].

BS-RNase multimers were firstly obtained upon subjecting the protein to incubation in 40% HAc followed by lyophilization [11], [12]. These conditions are known to extensively denature RNase A [46], with its oligomers forming only when the lyophilized powder is re-dissolved in “benign buffers” [46], like NaPi. Accordingly, one would expect similar results with BS-RNase when incubating either a pure isoform or any combination of them; therefore, for sake of clarity, all BS-RNase oligomers have been produced starting from the equilibrium mixture of the two native isoforms [6], i.e. M = M and MxM.

**Figure 1:**
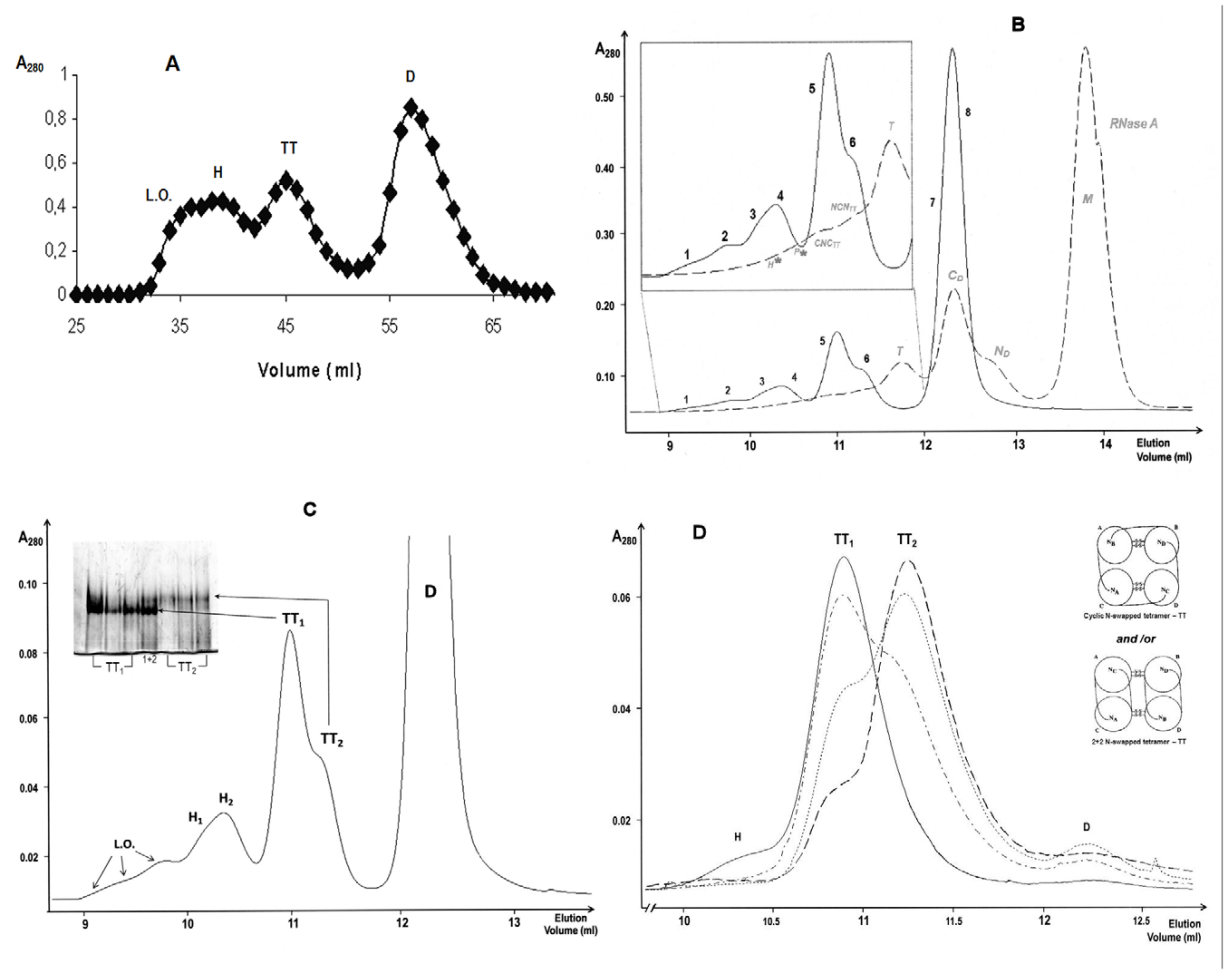
SEC chromatograms and PAGE under non denaturing conditions of BS-RNase aggregates obtained by lyophilising the protein from a 40% (v/v) acetic acid solution. (**A**) SEC pattern obtained with a Sephadex G100 column. Elution with ammonium acetate 0.1 M, pH 5.65, flow rate of 0.4 ml/min. (**B**) SEC chromatogram of BS-RNase multimers superimposed with that of RNase A oligomers: both patterns were obtained with a Superdex 75 10/300 GL column. Elution with 0.2 M NaPi, pH 6.7, flow rate 0.1 ml/min. (**C**) Enlarged Superdex 75 SEC pattern of BS-RNase aggregates; in the inset, 7.5% non denaturing PAGE of the two BS-tetramers, run-time 110 min. (**D**) Additional purification of the two BS-RNase tetramers: their mixture was concentrated to 25 µl in 0.4 M NaPi, and re-chromatographed in the Superdex 75 column equilibrated with the same buffer (dashed+dotted line). Then, TT_1_ and TT_2_ fractions were further purified: once for TT_1_, continuous line; twice for TT_2_, dotted and dashed lines, respectively. In the right part of the panel are reported the models of two N-swapped BS-RNase tetramers proposed by Adinolfi *et al.* [13]: they cannot be associated to both tetramers. The various BS-RNase species are: D, native dimer; TT_1_ and TT_2_, two tetrameric conformers, H (1 and 2), hexamers; L.O., larger oligomers. Concerning RNase A, grey italics labels: *M*, native monomer, *N_D_*, N-terminal-swapped dimer, *C_D_*, C-terminal-swapped dimer; *T*, trimers; *NCN_TT_*: double N+C-swapped tetramer; *CNC_TT_*: double C+N-swapped tetramer; P*: pentamers; H*: hexamers. The asterisk* is present to mention that P and H positions are derived from data obtained in [21].

Preliminary results obtained through size-exclusion chromatography (SEC) with a Sephadex G-100 column showed the presence of BS-RNase tetramers (TT), hexamers (H), and larger oligomers (L.O.) (Figure 1A). The chromatographic medium is crucial to purify different RNase oligomeric isoforms, but no improvement was achieved with a Superdex 200 column (*data not shown*). In contrast, a superior separation was obtained using a Superdex 75 10/300 GL column (Figure 1B, continuous line), reporting the yields of all BS-RNase oligomers in Table 1. We also attempted to refine the separation using two different cation-exchange columns, but, contrarily to RNase A [20], no conditions allowed us to improve the quality of the purification previously obtained with SEC (*data not shown*).

**Table 1:** Quantification and structural features of BS-RNase oligomers.

	Elution Volume (ml)		Yield (% of the total)		Hydrodynamic diameter (nm)^a^
BS-RNase species	WT	K113N	WT	K113N	WT
**D**	12.27±0.18	12.31±0.05	58.9±5.3	53.7±5.1	7.53±0.23
**TT_2/N_** b	11.19±0.04	11.31±0.09	10.2±2.2	12.2±1.0	8.51±0.32
**TT_1/C_** b	10.86±0.03	11.08±0.05	15.8±2.5	13.3±1.1	9.65±0.29
**H**	10.35±0.09	10.40±0.02	9.7±2.6	10.9±1.9	---
**L.O.** c	9.92±0.11	9.87±0.04	5.4±2.5	9.9±3.4	---

a Calculated from DLS analysis.

b The elution volumes of the BS-tetramers derive from their additional SEC purification with 0.4 M NaPi as eluent (see Figure 1D).

c L.O.: mixture of BS-RNase octamers and larger oligomers.

The BS-RNase pattern of Figure 1B is superimposed with the known [20] RNase A oligomerization profile (dashed line). By the way, we recall here that BS-RNase is a covalent dimer [3], while RNase A is natively monomeric. Therefore, the multimerization sequence of the seminal variant is dimer>tetramer(s)>hexamer(s)>octamer(s), etc, and accounts for the absence of BS-RNase peaks co-eluting with monomeric, trimeric and pentameric (data not shown, and [21]) RNase A. Nevertheless, the two RNases' profiles showed strong similarities: in fact, BS-RNase (native) dimer (fractions 7, 8, Figure 1B) eluted at the same volume of RNase A-C_D_ [20], [38]; in addition, a composite peak (fraction 5, 6) co-eluted with RNase A tetramers (NCN_TT_ and CNC_TT_ [20], in the grey magnification) and a composite peak (fractions 3, 4) overlaps the RNase A hexameric traces, whose position was determined in previous studies [21]; finally, also traces of larger BS-RNase multimers (octamers and so on) were clearly visible (fractions 1, 2). Each numbered fraction was separated, concentrated, and analyzed through 10% cathodic PAGE under non-denaturing conditions [47] (Figure S1), confirming that all peaks are BS-tetramers (TT, fractions 5, 6), hexamers (H, fractions 3, 4) and larger oligomers (L.O., fractions 1, 2), respectively, as reported in Figure 1C. Moreover, in panel C, at least two BS-hexamers (H_1_ and H_2_), and especially two different tetramers (TT_1_ and TT_2_) are visible. A 7.5% non-denaturing cathodic PAGE of tetramers (inset of Figure 1C) deriving from three preparations showed that BS-TT_1_ and -TT_2_ display different electrophoretic mobilities and shapes, with alterations in the charged groups exposure, or both [40]. Anyhow, although TT_1_ and TT_2_ are qualitatively different, they were not completely purified from each other. Thus, we collected their mixture, concentrated it in 0.4 M NaPi to the smallest volume possible, and re-chromatographed in the Superdex 75 column equilibrated with the same buffer (Figure 1D, dashed+dotted line). The two fractions collected were further purified and the separation was definitely satisfactory for TT_1_ (continuous line). Contrarily, it was not possible to completely purify TT_2_, even after two consecutive attempts (dotted and dashed lines, respectively). Nevertheless, the residual contaminant TT_1_ was only about 15% (dashed line), and the first part of TT_2_ peak was discarded for further analyses. Thus, also after this last purification the two tetramers confirmed to have different shape, and this fact is somehow surprising, considering the similar size, shape and charge exposure of the two BS-N-swapped tetrameric models proposed by Mazzarella and colleagues [13] and shown in the right part of Figure 1D: these two structures differ, in fact, only in the orientation of the N-swapped domains, and they are chromatographically undistinguishable. They represent the only possibility for BS-RNase to form different N-swapped tetramers, taking also into account the constraint given by the two disulfides involving Cys-31 and -32 that link the two subunits of the native dimer. Thus, a domain other than the N-terminus has to swap to justify the different behavior of BS-TT_1_ and -TT_2_, and the C-terminus can be the logical candidate to be swapped. This hypothesis is enforced by the evidence that, in SEC, RNase A-C_D_ partially precedes the N-dimer (N_D_, Figure 1B, dashed line), as BS-TT_1_ does towards BS-TT_2_. Consequently, BS-TT_1_ should be the C-swapped conformer, while BS-TT_2_ is assignable to one, or both, of the two N-swapped models proposed by Mazzarella and co-workers [13].

### Cross-linking of BS-RNase with DVS and DFDNB

**Figure 2:**
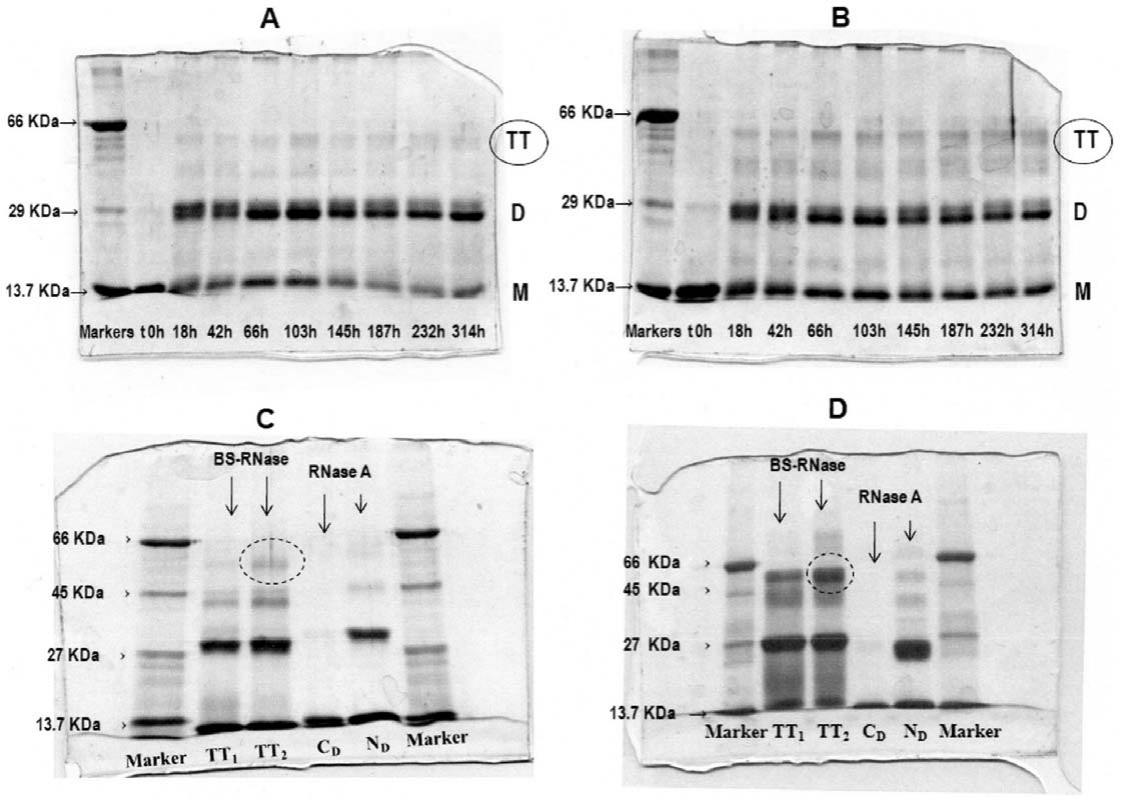
10% SDS-PAGE analysis of the crosslinking reactions of BS-RNase tetramers. (**A**), (**B**) DVS reaction time-course of TT_1_ and TT_2_, respectively. 8 µg of each aliquot-sample was electrophoresed after blocking the reaction with 0.2 M β-mercaptoethanol, final concentration. (**C**), (**D**) DFDNB reaction of BS-RNase tetramers and RNase A dimers analyzed under reducing and non-reducing conditions, respectively. Samples were concentrated to 1 mg/ml, and 10 µg of each were analyzed.

Divinylsulfone (DVS) cross-links BS-RNase His-12 and His-119 [48], the catalytic residues which lye close to each other at the active site. Being H12 and 119 at the N- and C-termini of the enzyme, respectively, DVS will yield oligomers that are stable under denaturing conditions only if 3D domain swapping had occurred [48]. Thus, BS-RNase tetramers were distinctly cross-linked with DVS, and the SDS-PAGE analysis of the reaction time-course is shown in Figure 2A,B. The reaction yield is low for both isomers, especially BS-TT_1_, because the conditions required for the reaction (sodium acetate, pH 5.0) were not optimal for oligomers' stability. Nevertheless, a light band corresponding to a MW comprised between 45 and 66 kDa, which includes a RNase tetramer (≈55 kDa), was visible for both isomers, indicating their domain-swapped nature. Furthermore, no higher M.W. bands are present in the panels, confirming that the two species analyzed are tetramers, and that no spuriously cross-linking due to occasional protein collisions occurred.

1,5-difluoro-2,4-dinitrobenzene (DFDNB) permits one to distinguish between N-terminal and C-terminal swapping because it selectively cross-links RNase Lys-7 and Lys-41 [49], thus stabilizing only the N-swapped oligomers. The cross-linking reaction was performed on both BS-RNase tetramers purified from SEC, and also on the positive and negative controls, RNase A-N_D_ and -C_D_, respectively. The reaction mixtures were analyzed by SDS-PAGE under reducing and non-reducing conditions (Figure 2C,D): a band of BS-TT_2_ corresponding to a cross-linked tetramer indicated by the dotted circle in both panels appears to be more intense than that of BS-TT_1_, either under reducing (panel C) or non-reducing (panel D) conditions. Whereas the results were not clear-cut, we can envisage that BS-TT_1_ contains fewer swapped N-termini than BS-TT_2_, because it has also to be considered that a significant tetrameric fraction dissociates before cross-linking occurs, and the cross-linking yield did not reach 100% even for the positive control, RNase A-N_D_. Thus, the DFDNB cross-linking appears helpful enough to support the hypothesis that BS-TT_1_ contains the C-terminal swapping, while BS-TT_2_ does not. We also cross-linked the native dimer to verify if the N-terminus lock could decrease or even delete the amount of N-swapping to occur. After SEC and cation-exchange purification [50] (Figure S2A,B and Discussion S1 file) the cross-linked protein was induced to oligomerize [11]. The resulting sample showed, after SEC purification (Figure S2C, continuous line), only one tetrameric peak, very probably the C-swapped one. Anyhow, the wideness and position of the peak (Figure S2C, and Discussion S1 file), cannot allow us to certainly assign it to the C-swapped isoform.

### Stability of the BS-RNase tetramers

**Figure 3:**
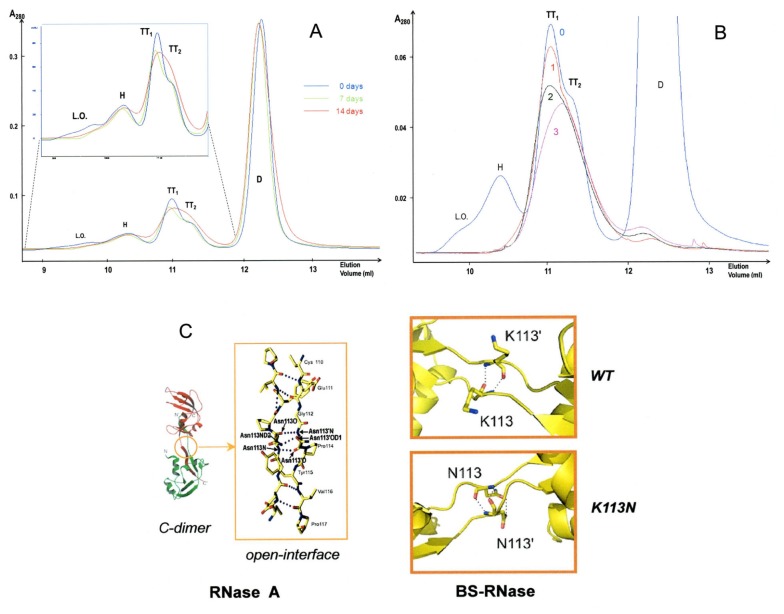
Analysis of the stability of BS-RNase oligomers. (**A**) SEC of the oligomers' mixture immediately dissolved in 0.2 M NaPi, pH 6.7 (day 0-blue curve), or after storing it at 4°C for one or two weeks (7 and 14 days, green and red curves), keeping constant the amount of the native dimer. (**B**) SEC of TT_1_ and TT_2_ gathered together immediately after their elution from the oligomers' mixture (day 0-blue curve, cfr. panel **A**) and re-cromatographed after a storage at 4°C in 0.2 M NaPi, pH 6.7, for one, two, or three days (1-red, 2-dark green, 3- pink curves, respectively). The SEC experiments were carried out with a Superdex 75 column. (**C**) Left panel, RNase A C-dimer [38], and (central panel) the open interface [51] stabilizing a C-swapped RNase A oligomer. Right panels, BS-RNase C-swapped open-interface: upper panel, wt (K113); lower panel, BS^K113N^ (N113 interplay, as in RNase A).

Considering that meta-stability hinders the characterization of BS-RNase tetramers, we studied their relative dissociation kinetics. Three SEC chromatograms obtained immediately after dissolving the lyophilized mixture in 0.2 M NaPi, pH 6.7 (blue curve), or storing at 4°C two aliquots of it for one or two weeks are shown in Figure 3A, green and red curves, respectively. The relative oligomers amounts changed, with BS-TT_1_ being less stable than BS-TT_2_, and this trend was confirmed by the chromatograms of the two tetramers, analyzed after keeping them isolated from the other oligomers, and incubated together at 4°C up to three days (Figure 3B).

In contrast to the extensively studied propensity of BS-RNase to swap its N-terminus [14]–[18], either the BS-C-terminus or the hinge loop linking it to the protein core had never been nowadays analyzed in detail. Several substitutions are present, in this region, with respect to RNase A [4]: in particular, G111, K113, and S115 of BS-RNase are, respectively, Glu, Asn and Tyr in RNase A [4]. In particular, the mutation at 113 can be crucial, because Asn-113 stabilizes the open-interface [51] of RNase A-C_D_, through the formation of a “steric zipper poly-Q-like” hydrogen bond network (Figure 3C, left and central panels) [38]. Consequently, the N113K substitution could destabilize the BS-RNase C-swapped oligomers (i.e. TT_1_), by deleting the intermolecular H-bond between the side chais of the two N113 [38], and possibly introducing electrostatic repulsion caused by the two complementary lysines side chains (Figure 3C, right panels). Anyhow, it has to be considered that the factors governing the interplay of four subunits are larger than those affecting the interactions of two protein bodies forming a dimer [50].

### Production and self-association of BS^K113N^ (K113N-BS-RNase)

**Figure 4:**
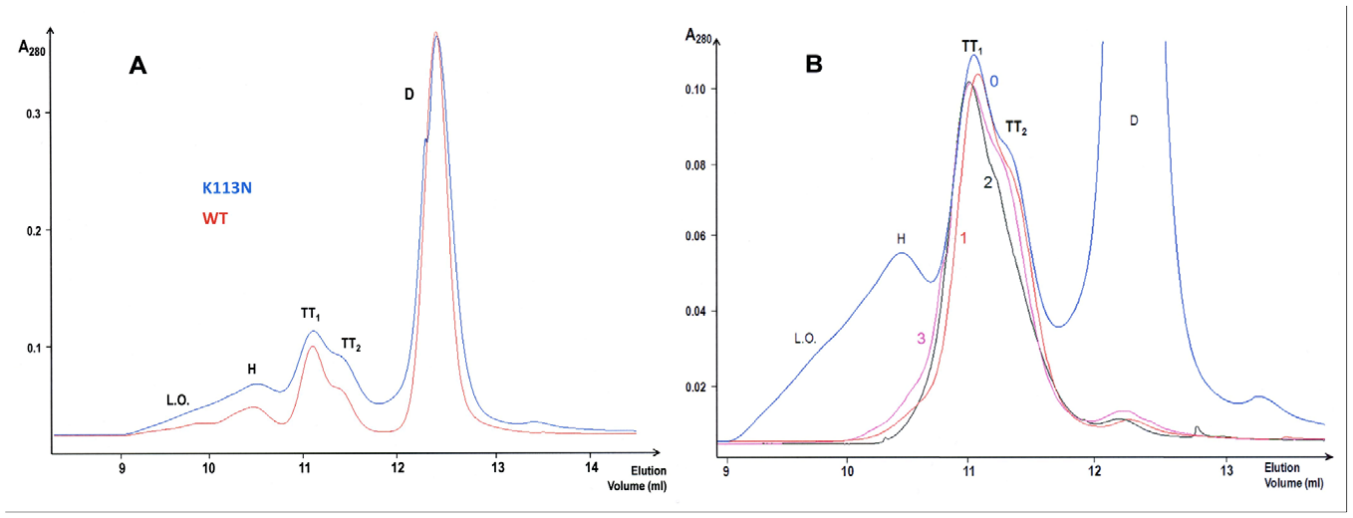
SEC chromatograms of K113N BS-RNase aggregates obtained by lyophilising the protein from a 40% (v/v) acetic acid solution. (**A**), Superdex 75 chromatogram of the mutant (blue) overlapped with a chromatogram of wt BS-RNase (red). (**B**), SEC of K113N BS-TT_1_ and TT_2_ gathered together immediately after their elution from the aggregates mixture (day 0-blue curve) and re-cromatographed after storage in 0.2 M NaPi, pH 6.7, for one, two, or three days (1-red, 2-dark green, 3-pink curves, respectively).

To evaluate the role of the 113 residue, a BS^K113N^ variant was produced, purified, and induced to oligomerize by the two methods [11], [35] used for the wt enzyme. The BS^K113N^ oligomerization induced by 40% HAc/lyophilization produced the results shown, and compared to those relative to wt, in Figure 4A and Table 1: it is clearly visible the higher aggregation yield of the mutant (53.7% of residual dimer), in particular of its larger oligomers (L.O.), with respect to the wt one (58.9% of dimer), as well as to that of RNase A, whose native residual monomer recovered after oligomerization is known to reach even 73% [52]. The two BS^K113N^ tetramers were withdrawn, concentrated and re-analyzed by SEC (Figure 4B), as was performed with wt tetramers (see Figure 3B), after one, two or three days. The results of Figure 4B reveal that, under these environmental conditions [52]–[55] BS^K113N^-TT_1_ is more resistant to dissociation than wt BS-TT_1_, indicating that the stability of this tetramer is increased by the K113N mutation. Finally, the two BS^K113N^ tetramers were cross-linked with DFDNB, under the same conditions used for the wt ones. The SDS-PAGE analysis (Figure S3) did not show significant differences from the results obtained with wt tetramers (see Figure 2C,D), indicating that the conditions required for the DFDNB reaction [49], [50] flatten the differences of stability related to the K113N mutation.

### Thermally-induced oligomerization of wt and BS^K113N^

The ‘thermal aggregation’ approach [35] can provide additional insights into the BS-RNase self-association mechanism, in particular into the possible swapping of its C-terminus. This possibility derives from the matter of fact that RNase A changes its propensity to swap its N- or C-terminus, or both, depending on the environmental conditions applied [35]. In particular, mildly denaturing conditions favor the exchange of RNase A N-terminus and, consequently, N_D_ formation, while more drastic conditions induce also the swapping of its C-terminus and formation of C_D_ [35].

**Figure 5:**
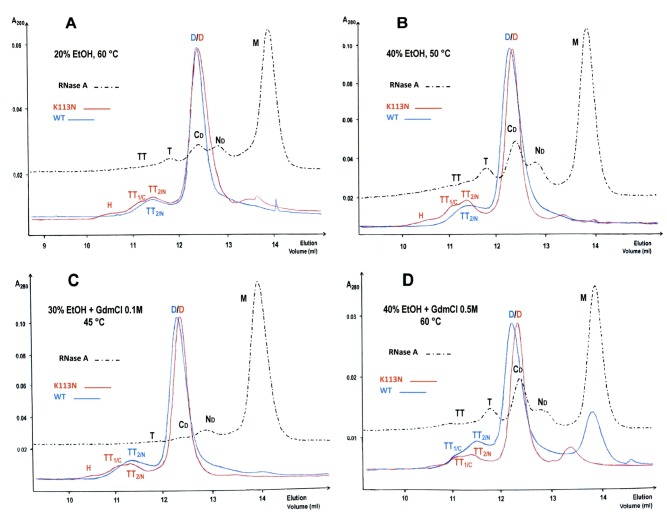
SEC profiles of wt and K113N BS-RNase aggregates obtained through thermal treatment. The various environmental conditions applied in aqueous solvents [35] are indicated in each of the **A–D** panels, in which the profiles obtained with the two BS-RNase variants (blue and red curves) are compared with the corresponding RNase A chromatograms (dotted black curves). Oligomers were obtained as follows: tubes containing 2.5–3.0 µl (0.5 mg) of each solution were put for 60 min in a thermostatically controlled bath at one of the temperatures indicated [35]. Then, 200 µl of 0.2 M NaPi, pH 6.7, heated to the same temperature of incubations [35], were added. Each sample was transferred to an ice-cold bath for 5 min, then injected onto a gel filtration Superdex 75 HR10/30 column. The different oligomers formed are labeled and correspond to the ones prepared by the lyophilization procedure (see Figure 1) [11]. GDMCl, guanidine hydrochloride; EtOH, ethanol.

The results of the experiments performed with BS-RNase are shown in Figure 5. Under all conditions applied for one hour, the only species of wt BS-RNase visible, besides the native dimer D, is TT_2_ (Figure 5A–C, blue curves). The only exception occurred when 40% aqueous EtOH contained 0.5 M guanidine (final concentration): in fact, after one hour incubation at 60°C, a little shoulder, assignable to BS-TT_1_, is also visible (Figure 5D, blue curve), although about half of the sample precipitated. On the contrary, RNase A showed to form, as expected [35], a C_D_ amount larger than N_D_ when it was incubated under the more drastic conditions (dotted black curves in A–D panels). Thus, again, the stability of BS-TT_1_ is definitely low, and here is additionally compromised by high temperature: consequently, we can consider the yields of BS-RNase tetramers as a balance between conditions severe enough to detach the protein terminals from the core and mild enough to avoid the dissociation of the newly formed oligomers [35], [53].

The results obtained, in parallel, with BS^K113N^ (red curves) show that its TT_1_ is definitely present in every aggregation profile, although its yield never surpassed that of TT_2_ (Figure 5B,D). Thus, the K113N mutation definitely favors BS-TT_1_ formation and indirectly confirms that this tetrameric conformer is formed through the C-terminus swapping. Consequently, the two BS-RNase tetramers will be called, from now on, TT_C_ and TT_N_, respectively, or also TT_1/C_ and TT_2/N_, as in Figure 5.

In addition, small but detectable amounts of BS^K113N^ oligomers larger than tetramers (L.O.) are visible in all chromatograms of Figure 5, especially in panel B (red curve). This result suggests that also BS large oligomers (L.O.), or some of them, contain C-swapped termini and that their formation was favored by the increase of the amount of TT_1/C_ [35].

Finally, the higher the denaturing strength of the medium (i.e., containing guanidine), the lower the temperature necessary to avoid a partial protein precipitation. In fact, substantially equal amounts of BS^K113N^-TT_1/C_ and -TT_2/C_ formed (Figure 5C, red curve) when the temperature decreased from 60 to 45°C, while, instead, this ‘cooling’ event favored RNase A-N_D_ over -C_D_ (dotted black curve). Taken together, all these final observations suggest that the optimal conditions to induce BS-RNase thermal aggregation are slightly milder than those promoting the same event in RNase A [35], [53].

### Dynamic Light Scattering (DLS) and Molecular Modeling of BS-TT_1/C_

DLS, which measures the hydrodynamic diameter of a protein [56], can be useful to obtain informations about the disposition of the BS-tetramers' subunits, in particular the differences given by N- and/or C-swapping. The data obtained (Table 1) show that the hydrodynamic diameter of BS-RNase native dimer is in agreement with the value relative to its crystallographic structure(s) [57], [58]. Instead, the BS-TT_2/N_ diameter is slightly larger and consistent with the two cyclic N-swapped-only models proposed by Adinolfi *et al.* [13]. Finally, as expected from SEC (see Figure 1C), the diameter measured for BS-TT_1/C_ is larger than the BS-TT_2/N_ one, and cannot fit the cited models.

**Figure 6:**
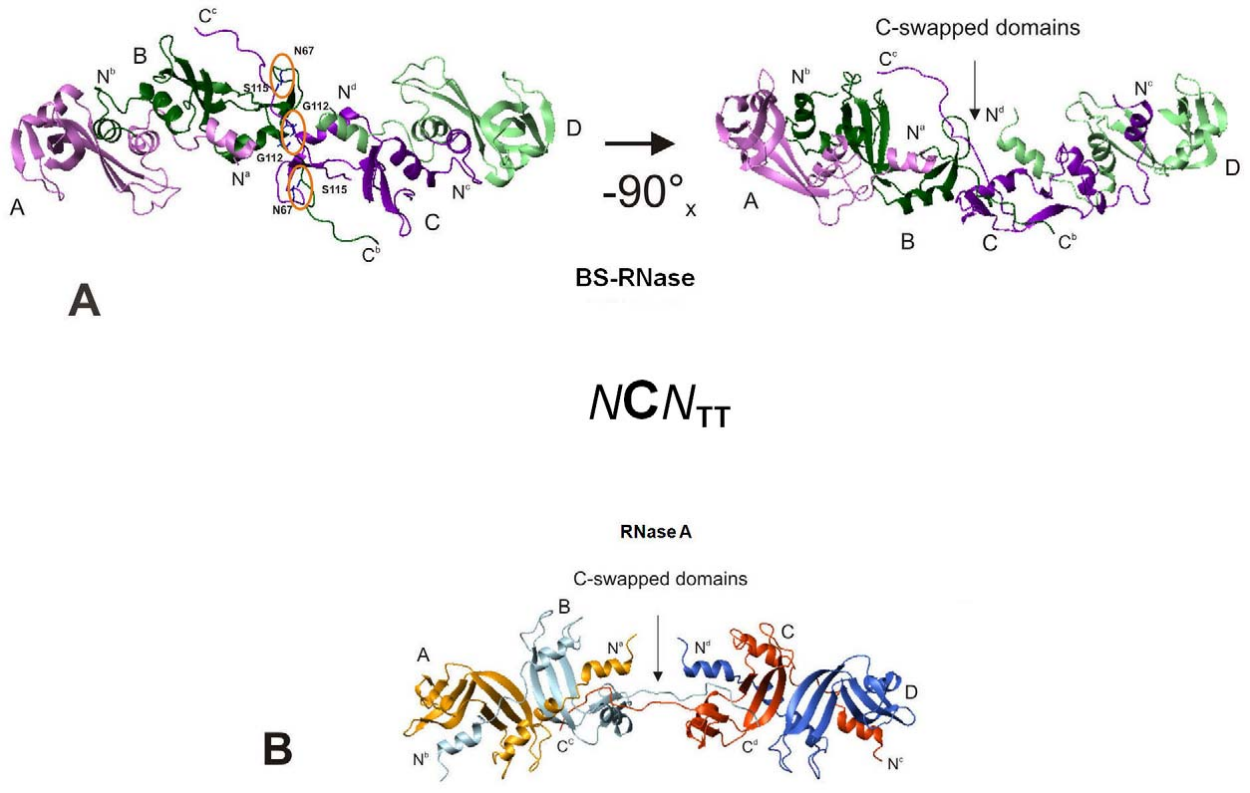
Molecular docking models of *N*C*N*-swapped BS-RNase and RNase A. (**A**), *left panel*, “quasi-linear” [40] *N*C*N*_TT_ model: the stabilizing intermolecular H-bonds between the two central subunits are indicated with orange circles; *right panel*, the same structure rotated 90° around the x-axis. (**B**) modeled structure of RNase A *N*C*N*_TT_ [40], [41] reported for comparison.

Thus, on the basis of DLS data, the known structure of MxM BS-dimer [57], and the structural constraints imposed by the inter-subunit disulfides, we modelled the structure of BS-TT_1/C_. Models were built by molecular docking, starting from two identical dimers of wt N-swapped BS-RNase (MxM). Each dimer had one C-terminus “opened”, with a conformation based on the crystal structure of the C-swapped RNase A (PDB code 1F0V) [38]. Then, fifty putative tetramers were generated and, by the docking and energy minimization algorithms, three representative low energy structures were selected and here shown in Figures 6 and S4. The structure which best fits TT_1/C_ hydrodynamic diameter (Table 1) is the “quasi-linear” [40] one, visible in two different orientations in the right and left panels of Figure 6A. This model can also explain the chromatographic differences existing between BS-TT_1_ and -TT_2_ (see Figure 1, Table 1), and consists of two native N-swapped dimers (MxM) linked by the swapping of the C-termini of their central subunits (dimer-C-*swap*-C-dimer). Thus, this tetramer globally swaps four N-termini and two central C-termini, and can be named also BS-NCN_TT_ [20]. Its C-swapped open interface [51] is stabilized by two inter-subunit H-bonds forming between the two Gly-112, and between Asp-67 and Val-116 of the two central complementary subunits (left panel of Figure 6A). This structure is different from the one of RNase A NCN_TT_ [40], [41] reported in Figure 6B for comparison, although the swapping sequences of the two tetramers are identical: in fact, the structural differences existing between of RNase A-N_D_ [37] and BS-RNase [57] induce different structures in the tetramers. In Figure S4A,B two alternative BS-NCN_TT_ models, increasingly bent with respect to the structure of Figure 6A, are shown. These structures lack the Gly-112 and Asp-67/Val-116 H-bonds of the “quasi-linear” model, and their hydrodynamic diameters are slightly different from the one measured for TT_1/C_ with DLS (Table 1), although they represent two other energy minima.

### Biological activities of BS-RNase oligomers

#### Enzymatic assays

Native BS-RNase is known to cleave double-stranded RNA (dsRNA), as well as the pool of its multimers does [12], although the activity of the latter has not been studied in detail yet. Instead, it is well-known that RNase A oligomers acquire the ability to degrade double-stranded polyribonucleotides, with the C-swapped species being more active than the corresponding N-swapped conformers [20].

Thus, we tested dimers and oligomers of both wt and BS^K113N^ on yeast RNA and poly(A)•poly (U), i.e. single-stranded (ss) and dsRNA, respectively (Table 2). The data obtained on yeast RNA confirmed that dimers and multimers of both BS-RNase variants are active, but somewhat less than monomeric RNase A [12], [20] used here as standard. On the contrary, the multimers of both BS-variants were more active against poly(A)•poly(U), with respect to their corresponding native dimer. Interestingly, the specific activity definitely increased from BS-dimer to tetramers, but only a slight activity augment was observed for the oligomers larger than tetramers (L.O.). Anyway, either wt or BS^K113N^ TT_1/C_s were slightly more active than the corresponding TT_2/N_s. This behavior qualitatively parallels the one of RNase A oligomers ([20] and Table 2), and can be ascribed to a higher basic charge density or exposure [2] depending on C-swapping event(s) rather than on N-swapping(s) [20].

**Table 2:** Enzymatic and cytotoxic properties of BS-RNase oligomers.

	Enzymatic Specific Activity, 23°C [20]				Cytotoxic Activity			
	Yeast RNA (Kunitz units/mg enzyme)		poly (A) : poly (U) (units/mg enzyme)		IC_50_ a (µg/ml)		IC_50_ Potentiation Factor^b^	
BS-RNase species	WT	K113N	WT	K113N	WT	K113N	WT	K113N
**D**	14.8±2.5	13.2±1.8	9.3±0.9	7.8±0.4	155±9.4	>240	1	1
**TT_2/N_**	10.1±2.0	10.3±1.6	29.4±1.8	28.6±2.3	13.2±0.6	65.6±5.1	11.7	>3.7
**TT_1/C_**	12.3±2.3	11.7±1.8	36.1±1.2	33.1±1.5	17.2±1.0	64.9±5.9	9.0	>3.7
**L.O.** c	10.3±2.6	9.8±2.0	38.0±3.1	37.3±1.5	9.1±0.5	38.1±2.7	17.0	>6.3

*RNase A species*	WT		WT
***M***	106.2±1.4		1.6±0.1
***N_D_***	70.3±1.8		3.1±0.1
***C_D_***	63.3±1.6		12.2±0.6
***NCN_TT_***	36.4±2.1		26.8±3.1

a IC_50_ mean values (± S.D.) from three independent experiments on VIT1 cells after 72 h.

b folds vs the IC_50_ value of native BS-RNase dimer D.

c L.O.: mixture of BS-RNase hexamers, octamers, and larger oligomers.

Finally, the BS-dimer of wt was slightly more active than the K113N one (Table 2). Contrarily, the activities displayed by the homologous wt or BS^K113N^ multimers were almost comprised within the experimental error, indicating that the loss of K113 positive charge did not significantly affect the catalytic activity of BS-RNase oligomers. Considering that dsRNAase activity increases with the positive charge density of the active site region [2], [20], we can envisage that the charge of K113 side chain does not affect RNase-dsRNA recognition.

#### Cytotoxicity assays

It was mentioned before that native BS-RNase displays several biological actions, especially a potentially therapeutic cytotoxic activity [7], [8], owned by only the N-swapped (MxM) dimeric isoform [9], [10]. Thus, on the basis of the enzymatic activities reported (Table 2), we evaluated if BS-RNase multimerization could affect the cytotoxic potential of the native dimer.

**Figure 7:**
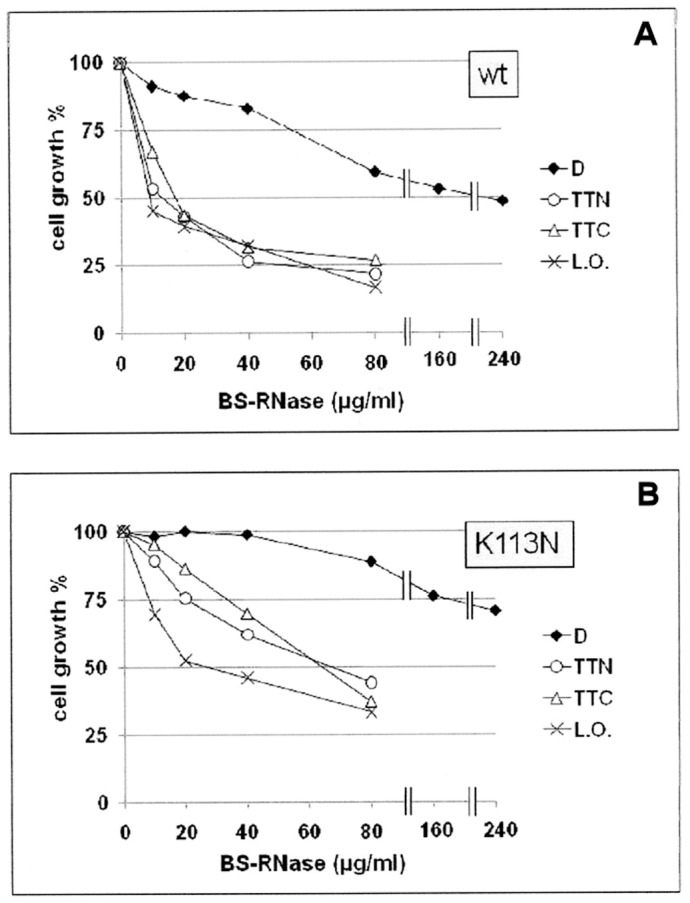
Action of BS-RNase oligomers on the proliferation of mesenchimal VIT1 cells. (**A**) wt, and (**B**) K113N BS-RNase. Cells were cultured in RPMI 1640 medium supplemented with 2 mM glutamine, 10% FBS, and 50 µg/ml gentamicin sulphate. After addition of the BS-RNase species, 10 to 240 µg/ml of BS-RNase (native) dimer, or of 10 to 80 µg/ml of BS TT_1/C_, TT_2/N_, or a mixture of larger oligomers (L.O.), cells were incubated for 72 h at 37°C with 5% (v/v) CO_2_. At the end of the treatments cells were stained with a Crystal Violet solution and the survival was measured, and compared to the control lacking any RNase species, as reported in Materials and Methods. Experiments were performed in triplicate; the S.D. are comprised between 4.5 and 6.0% for wt (**A**), and between 5.1 and 7.7% for K113N BS-RNase (**B**).

To avoid to collect data derived by genetic alterations of immortalized or tumor cell lines, the primary mesenchymal cell line VIT1 was chosen here as a model to analyze the inhibitory effect of both BS-RNase variants on cell growth. Cells were treated with increasing concentrations of dimer (D), or of TT_1/C_, TT_2/N_, or a mixture of BS-L.O.. In Figure 7, it is clearly visible that both wt and BS^K113N^ multimers display a cytotoxic activity higher than the corresponding dimers, in parallel with the enzymatic activity trend [20], [42], and in line with previous data obtained with RNase A oligomers [42]. In particular, Table 2 reports that wt and BS^K113N^ tetramers' IC_50_ values, obtained from growth inhibition curves shown in Figure 7, are about 10-fold, and more than 3.7-fold, lower than those of their native dimers, respectively. In addition, our data show that, within the same BS-RNase species, the two tetramers display an activity very similar to each other, and that BS-RNase larger oligomers (L.O.) decrease the IC_50_ value 17-fold for wt, and more than 6.3-fold for BS^K113N^, relative to their dimers, respectively (Figure 7, Table 2). Finally, and notably, the present data become additionally intriguing by observing that VIT1 cells are substantially resistant to RNase A native monomer or multimers, i.e. N_D_, C_D_, CNC_TT_, NCN_TT_, whose structural features have been previously described [20], and/or also to a mixture of larger RNase A oligomers (L.O.), as it is shown in Figure S5.

Altogether, these results indicate a potential therapeutic role for BS-RNase multimers, support the idea that the increase of cytotoxicity induced by RNase multimerization is correlated to the augmented enzymatic activity versus dsRNAs or dsRNA/DNA hybrids, but not towards ssRNA [20], [42], and suggest also that BS-RNase oligomers, being meta-stable, are active on the mentioned RNAs during the initial phases [59] of gene transcription.

### Concluding remarks

The results presented in this work elucidate several structural features underlying BS-RNase self-association through double domain swapping [19], providing, for the first time, experimental evidence for C-terminus swapping to occur in this protein, and the functional consequences related to it. Furthermore, the seminal enzyme displays to multimerize to a higher extent than RNase A does (see Table 1).

In addition, and finally, the present work confirms that 3D domain swapping event, although not cleared in all its aspects, such as its predictability, is a very intriguing phenomenon often associated to the incoming or settling of important biological actions. In this context, RNases continue to represent interesting models [20], [55], [60]–[62] to study protein aggregation through domain swapping, and suggest that, when not leading to fibrillization, a controlled protein self-association [31], [32], [55] can be advantageous in terms of acquired or increased potentially therapeutic biological activities, as it is for BS-RNase.

## Materials and Methods

### Materials

The QuikChange Site-Directed Mutagenesis Kit and protocol (Stratagene, La Jolla, CA, USA) were used to produce the BS-RNase mutants, starting from the pET-22b (+) plasmid cDNA coding for the N67D-BS-RNase variant. The N67D substitution prevents the spontaneous deamidation of Asn-67 in the native protein, which should consequently be isolated as a mixture of Asn, Asp and isoAsp variants at position 67 [44]. The biological activity and the structural features of the three derivatives are almost identical to those of the native enzyme [45], thus we used the N67D variant to avoid side-reactions, and referred to as wild-type (wt).

As for the (N67D/)K113N-BS-RNase variant, henceforth named BS^K113N^, the mutagenic primers were 5′**-**GCTTGTGGCGG**TAA**CCCGTCCGTGCC-3′, and 3′-GGCACGGACGGGTTACCGCCACAAGC-5, confirming the mutations by DNA sequencing. The mutant was produced and purified from *E. coli* as a monomer, with Cys-31 and 32 linked to two glutathione molecules [15]. After selectively reducing the mixed disulfides, they were either carboxyamidomethylated with iodoacetamide [63] to obtain monomers for CD analysis, or dialyzed against 0.1 M Tris/acetate pH 8.4 followed by SEC onto a Sephadex G-75 column to obtain dimers [64]. The protein solution was incubated at 37°C for at least 72 h to reach the equilibrium between the two MxM and M = M isoforms [6]. The same protocol was used to express and purify N67D-BS-RNase (wt), while both recombinant dimeric BS-RNases were treated with *Aeromonas proteolytica* aminopeptidase (Sigma) [65] to remove Met-1 before cytotoxicity arrays.

RNase A (R5500, type XII A), used here as a standard, and poly (A)•poly (U), were purchased from Sigma; yeast RNA was purchased from Boehringer.

### Production of BS-RNase oligomers

To prepare BS-RNase oligomers we followed two different procedures: in the “classic” aggregation protocol [11], 5 to 50 mg/ml protein samples dissolved in 40% HAc were lyophilized and re-dissolved in 0.2 M, or 0.4 NaPi, pH 6.7 [20], [46]. As for the thermally-induced oligomerization procedure, some of the conditions used elsewhere [35], [53] were chosen in this work. Half mg BS-RNase samples were dissolved at 150–200 mg/ml in various aqueous media (See Results and Discussion and Figure 5) and heated to 45, 50, or 60°C up to 1 h. At the end of each treatment, a 80–100 fold excess of 0.2 M NaPi, pH 6.7, pre-heated at the incubation temperature [35], was added. Each mixture was brought to 0–4°C, and chromatographed through SEC.

### Chromatographic purification and quantification of the BS-RNase oligomers

Purification and analyses of the RNase oligomers were performed through SEC using a Sephadex G-100 column (70×1.5 cm), flow rate 0.4–0.5 ml/min, or with Superdex 75 or 200 10/300 GL columns (GE-Healthcare) attached to an ÄKTA FPLC system (GE-Healthcare), flow rate 0.08–0.10 ml/min [20], at room temperature. The purified oligomers were kept at 4°C until use, or concentrated with Millipore Centricon Ultra-filters (C.O. 10 kDa) just before use.

BS-RNase multimers were chromatographed also through a Source 15S HR10/10 or Mono-S cation-exchange columns (GE-Healthcare): elution was performed with a 0.09–0.20 M NaPi gradient, pH 6.7 [20]; flow rate was between 0.4 and 1.2 ml/min. Additional experiments were performed different buffers and/or gradients: start from 0.10, 0.15, or 0.20 M NaPi, gradient to 0.40 M NaPi, pH 6.7; or, finally, 0.10 M NaPi, pH 6.7, with a 0.05 to 0.40 M NaCl gradient.

The concentration of BS-RNase and RNase A species was spectrophotometrically measured at 278 nm with a ε^1%^_278_ of 4.65 [66], and at 280 nm, ε^1%^_280_ of 7.3 [67], respectively. Each RNase oligomer amount was measured also as the percent area of its SEC peak relative to the sum of the areas of all peaks eluted. The values reported are means of five to eight measurements.

### Cross-linking

Cross-linking with divinylsulfone (DVS) was performed following the method of Ciglic *et al.* [48], with slight modifications: the reaction was performed for 315 h at 20°C, not 30°C [48], to minimize the possible tetramers' dissociation. At the chosen times, aliquots of 20 µg of the protein were withdrawn to quench the reaction by adding β-mercaptoethanol, to a final concentration of 0.2 M. Each aliquot was kept at 4°C, until 8 µg of it were subjected to SDS-PAGE.

1,5-difluoro-2,4-dinitrobenzene (DFDNB) was used by using the protocol of Lin *et al.* [49], with some modifications in order to limit a contemporary undesired oligomer's dissociation. 0.2 mg/ml of each oligomeric RNase species were dissolved in 0.1 M NaPi, and brought to pH 8.0 with few microliters of Na_2_B_4_O_7_. Four microliters of DFDNB, 0.37 mM in 2% (v/v) methanol solution, were added every 8–10 min, over a 3 h period, to the RNase species separately kept and stirred in the dark at 8°C, to a final molar RNase/DFDNB ratio of about 1∶2. The stirring was protracted for additional 20 h, and samples were finally concentrated to 1 mg/ml to be analyzed through SDS-PAGE. The same procedure was followed for BS-RNase native dimer, but in 50 mM borate buffer, pH 8.5, at room temperature [49], but additionally stirring in the dark for not more than 4 h to limit protein precipitation.

### Gel electrophoresis

SDS-PAGE (10, 12.5, or 15% polyacrylamide gel, Tris/glycine buffer, pH 8.3) was performed at 20 mA, for 70–120 min, depending on % of polyacrylamide, at room temperature.

Cathodic PAGE under non-denaturing conditions was performed according to Goldenberg [47], with slight modifications, using a pH 4.0 β-alanine/HAc buffer. 7.5, 10, or 12.5% polyacrylamide gels were run at 20 mA for 60–100 min, at 4°C, fixed with 12.5% trichloroacetic acid and stained with 0.1% aqueous Coomassie brilliant blue.

### Dynamic Light Scattering (DLS)

DLS measurements were performed following the procedures described in [68], and the data processed, on a Zetasizer Nano-S device from (Malvern Instruments) to measure the hydrodynamic diameter of the BS-RNase species dissolved in NaPi 0.2 M, pH 6.7. The temperature of the sample was controlled by a thermostat to within ±0.1°C. The solution was filtered with “Anotop” filters immediately before use and 12.5×45-mm disposable cells equipped with stopper were used.

### Molecular modelling and docking

The structures of the C-swapped dimeric wt BS-RNase and BS^K113N^ were modelled starting from the NMR structure of the monomeric BS-RNase derivative (mBS, PDB code 1QWQ [15]), and from the RNase A C-dimer crystallographic structure (PDB code 1F0V [38]), respectively. The mentioned atomic coordinates were used as a template to predict the 3D structure of the variants, using the Modeller 9.9 program [69], and the score of variable target function method [70] to evaluate the quality of the models. BS-RNase tetrameric models were built by docking using GRAMM-X (http://vakser.bioinformatics.ku.edu/resources/gramm/grammx). The starting structures used for the docking were two BS-RNase MxM dimers (PDB code 1BSR [57]). In each of them, the same Φ and ψ dihedral angles of the RNase A C-dimer crystallographic structure (PDB code 1F0V [38]) were imposed to the C-terminal residues 110–124. To perform the docking it was imposed that the interface of each dimer included the 111–113 residues of the C-swapped chain(s). The quality of the tetramers so obtained was checked with ANOLEA [71], and all the structures were virtualized with PYMOL [72].

### Biological assays

#### Enzymatic assays

The enzymatic activities of the BS-RNase and RNase A species were measured at 23°C [20], using a thermostatically controlled Beckman DU-650 spectrophotometer. Assays with yeast RNA as a substrate were performed using 0.5 µg of each RNase (BS or A) species at 300 nm, according to the method of Kunitz [73]. Assays with dsRNA poly(A)•poly(U) were performed, as described in [12], at 260 nm, with 5 µg of BS-dimer, and 2 µg of each BS-tetramer (TT_1_ or TT_2_), and of larger oligomers (L.O.). Concerning RNase A, the amounts used were: monomer, 12 µg; dimers (N_D_ or C_D_), 5 µg; tetramers (NCN_TT_ or CNC_TT_), 2 µg. The Abs_260_ and Abs_300_, respectively, were recorded versus time, and the specific activity of the various RNase species was calculated using the following equation: (ΔAbs/time (min))/amount of enzyme (mg). All the enzymatic activity values are means of three different assays ± S.D.

#### *In vitro* cytotoxicity assays

The primary pancreatic mesenchymal cell line VIT1 (Chemicon International, Milan, Italy) was grown in RPMI 1640 supplemented with 2 mM glutamine, 10% FBS, and 50 µg/ml gentamicin sulfate (BioWhittaker, Lonza, Bergamo, Italy) at 37°C with 5% CO_2_.

Cells were seeded in 96-well plates (2.5×10^3^ cells/well), then treated 24 hours later with the various protein species, and further incubated for 72 h. At the end of the treatment, cells were stained with a Crystal Violet solution (Sigma, Milan, Italy). The dye was solubilised in PBS containing 1% SDS and spectrophotometrically measured (Abs_595 nm_) to determine cell growth. IC_50_ values were obtained (mean ± S.D.), and represent the concentration of the various compounds when 50% growth inhibition is recorded. Three independent experiments were performed for each assay condition.

## Supporting Information

Figure S1 **Native PAGE of BS-RNase species eluted from SEC visible in** **Figure 1B** **.** 10% PAGEs under non denaturing conditions [47] were performed with the BS-RNase species eluted from SEC and concentrated to 0.6–0.7 mg/ml, in NaPi 0.1 M pH 6.7. (**A**) Only the fractions corresponding to tetramers (5 and 6) and dimer (7 and 8) were analyzed, together with the mixture (Mix) of the aggregates not separated through SEC (right lane). In this lane, 5 µg of RNase A monomer, less cationic and with a lower mobility than BS-RNase native dimer, were also added. Run-time 80 min; (**B**) Also the BS-RNase oligomers larger than tetramers are analyzed: electrophoresis was extended for 110 min, and the dimer D almost escaped out from the gel (lanes 7 & 8), but more than one tetrameric (TT, lanes 4, 5 & 6) and hexameric (H, lanes 3 & 4) conformers are present. Finally, more than one octamer and/or larger oligomers (L.O., lanes 2 & 3) are probably present, while only a light smear is visible in lane 1. The ‘Mix’ does not contain here RNase A.
(TIF)

Figure S2 **Oligomerization pattern of wt BS-RNase after cross-linking of the native dimer with DFDNB.** (**A**) The cross-linked protein was first purified with Superdex 75 column (dotted line) obtaining four main fractions (*1–4*). Each fraction was separately induced to oligomerize from 40% HAc solutions [11]. The result obtained with fraction ***3*** (continuous line) is reported together with the pattern relative to an aliquot of BS-RNase that was not cross-linked (dashed line). The pattern of the cross-linked protein shows the presence of both tetramers, and also a badly resolved portion of larger oligomers. Flow rate 0.08 to 0.10 ml/min, injected volume 25 µl. (**B**) Further purification of DFDNB-BS-fraction ***3*** through a cation-exchange column Source 15S HR 10/10: the two patterns obtained under the two conditions chosen (100 and 150 mM NaPi, pH 6.7) to better fix the protein to, and elute it from, the resin are shown in blue and red lines, respectively. The linear gradient applied to rise NaPi concentration from 0.10 or 0.15 M up to 0.40 M was applied after 20 ml (three column volumes) from the elution start. Gradient time-course: blue curves, 75 min; red curves, 62.5 min; flow rate, 1.2 ml/min. Continuous lines, DFDNB-BS-RNase-fraction ***3*** (panel A); dashed lines, native dimeric BS-RNase. The DFDNB-BS portion(s) preceding the dashed+dotted vertical lines (limit to avoid contamination of un-reacted BS-RNase, see dashed line-patterns of native BS-RNase) were collected, desalted, concentrated and induced to oligomerize through lyophilization from 40% HAc solutions [11]. (**C**) The resulting mixture was analyzed through SEC, Superdex 75 column: continuous line, sample purified through SEC+cation-exchange (panels A+B) before inducing its oligomerization; dashed line, sample purified only with SEC (same pattern of panel A, continuous line), reported for comparison. Flow rate 0.08 to 0.10 ml/min, injected volume 25 µl.
(TIF)

Figure S3 **10% acrylamide SDS-PAGE of BS^K113N^ tetramers after their cross-linking with DFDNB.** The lane corresponding to TT_2_ (considered totally N-swapped) shows a slightly higher amount of cross-linked products than the corresponding TT_1_.
(TIF)

Figure S4 **Alternative bent NCN_TT_ models for TT_1/C_.** The modeled structures (**A**,**B**) display an increasing central bending with respect to the one shown in Figure 6A, and represent energy minima as well as the latter, but their hydrodynamic diameter is less in agreement with the one experimentally measured for BS-TT_1/C_ (Figure 1, Figure 5).
(TIF)

Figure S5 **Action of RNase A oligomers on the proliferation of mesenchimal VIT1 cells.** Cells were cultured in RPMI 1640 medium supplemented with 2 mM glutamine, 10% FBS, and 50 µg/ml gentamicin sulphate. After the RNase A species addition, 40 to 240 µg/ml, cells were incubated for 72 h at 37°C with 5% (v/v) CO_2_. At the end of the treatments cells were stained with a Crystal Violet solution and the survival was measured and compared to the control without any RNase species. RNase A species: M, monomer; DN, N-swapped dimer; DC, C-swapped dimer; TTN, NCN-swapped tetramer; TTC, CNC-swapped tetramer; L.O., mixture of RNase A pentamers, hexamers, and larger oligomers.
(TIF)

Discussion S1 **Discussion concerning the results derived from the patterns reported in Figure S2.**
(DOC)
