# Supplementary figures and images for: Double Domain Swapping in Bovine Seminal RNase: Formation of Distinct N- and C-swapped Tetramers and Multimers with Increasing Biological Activities
Source: PLoS One. 2012 Oct 11;7(10):e46804. doi: 10.1371/journal.pone.0046804 (PMC3469567; doi:10.1371/journal.pone.0046804)

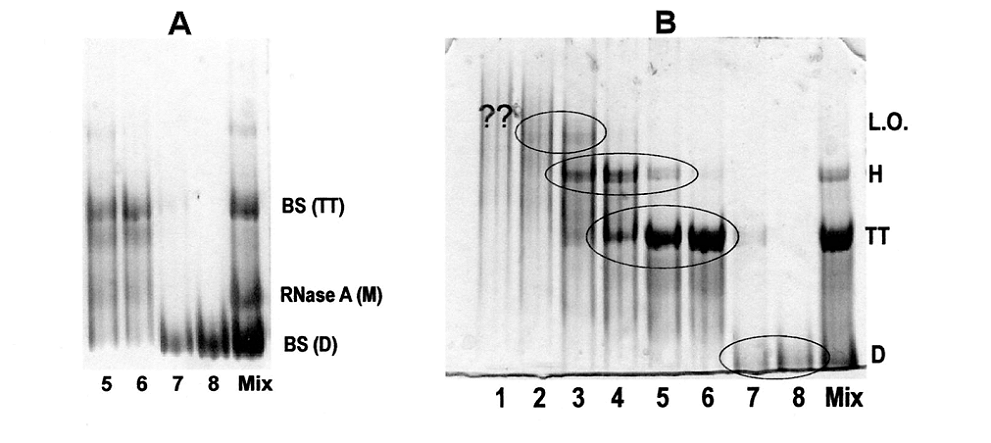

Supplement: Figure S1 — Native PAGE of BS-RNase species eluted from SEC visible in Figure 1B . 10% PAGEs under non denaturing conditions [47] were performed with the BS-RNase species eluted from SEC and concentrated to 0.6–0.7 mg/ml, in NaPi 0.1 M pH 6.7. (A) Only the fractions corresponding to tetramers (5 and 6) and dimer (7 and 8) were analyzed, together with the mixture (Mix) of the aggregates not separated through SEC (right lane). In this lane, 5 µg of RNase A monomer, less cationic and with a lower mobility than BS-RNase native dimer, were also added. Run-time 80 min; (B) Also the BS-RNase oligomers larger than tetramers are analyzed: electrophoresis was extended for 110 min, and the dimer D almost escaped out from the gel (lanes 7 & 8), but more than one tetrameric (TT, lanes 4, 5 & 6) and hexameric (H, lanes 3 & 4) conformers are present. Finally, more than one octamer and/or larger oligomers (L.O., lanes 2 & 3) are probably present, while only a light smear is visible in lane 1. The ‘Mix’ does not contain here RNase A. (TIF) [file pone.0046804.s001.tif]

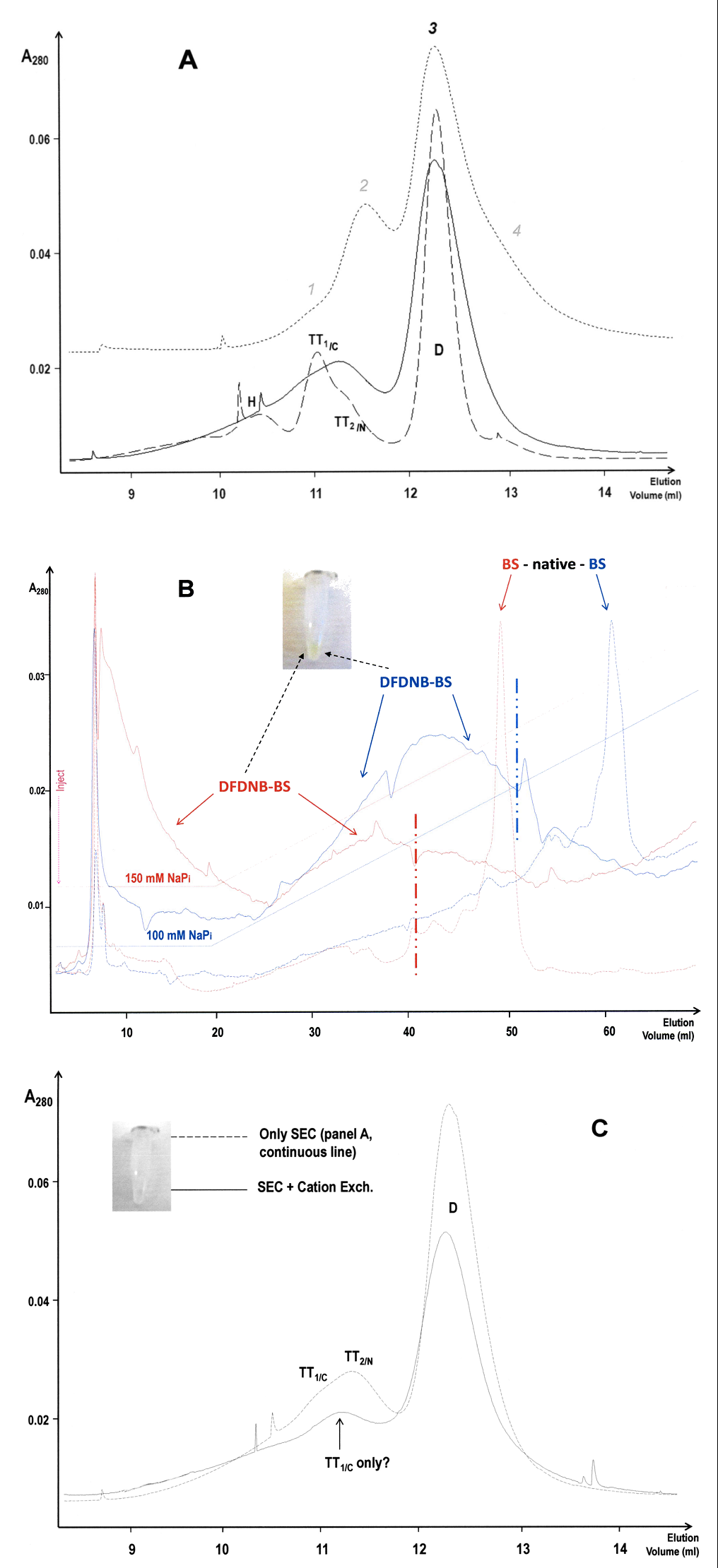

Supplement: Figure S2 — Oligomerization pattern of wt BS-RNase after cross-linking of the native dimer with DFDNB. (A) The cross-linked protein was first purified with Superdex 75 column (dotted line) obtaining four main fractions (1–4). Each fraction was separately induced to oligomerize from 40% HAc solutions [11]. The result obtained with fraction 3 (continuous line) is reported together with the pattern relative to an aliquot of BS-RNase that was not cross-linked (dashed line). The pattern of the cross-linked protein shows the presence of both tetramers, and also a badly resolved portion of larger oligomers. Flow rate 0.08 to 0.10 ml/min, injected volume 25 µl. (B) Further purification of DFDNB-BS-fraction 3 through a cation-exchange column Source 15S HR 10/10: the two patterns obtained under the two conditions chosen (100 and 150 mM NaPi, pH 6.7) to better fix the protein to, and elute it from, the resin are shown in blue and red lines, respectively. The linear gradient applied to rise NaPi concentration from 0.10 or 0.15 M up to 0.40 M was applied after 20 ml (three column volumes) from the elution start. Gradient time-course: blue curves, 75 min; red curves, 62.5 min; flow rate, 1.2 ml/min. Continuous lines, DFDNB-BS-RNase-fraction 3 (panel A); dashed lines, native dimeric BS-RNase. The DFDNB-BS portion(s) preceding the dashed+dotted vertical lines (limit to avoid contamination of un-reacted BS-RNase, see dashed line-patterns of native BS-RNase) were collected, desalted, concentrated and induced to oligomerize through lyophilization from 40% HAc solutions [11]. (C) The resulting mixture was analyzed through SEC, Superdex 75 column: continuous line, sample purified through SEC+cation-exchange (panels A+B) before inducing its oligomerization; dashed line, sample purified only with SEC (same pattern of panel A, continuous line), reported for comparison. Flow rate 0.08 to 0.10 ml/min, injected volume 25 µl. (TIF) [file pone.0046804.s002.tif]

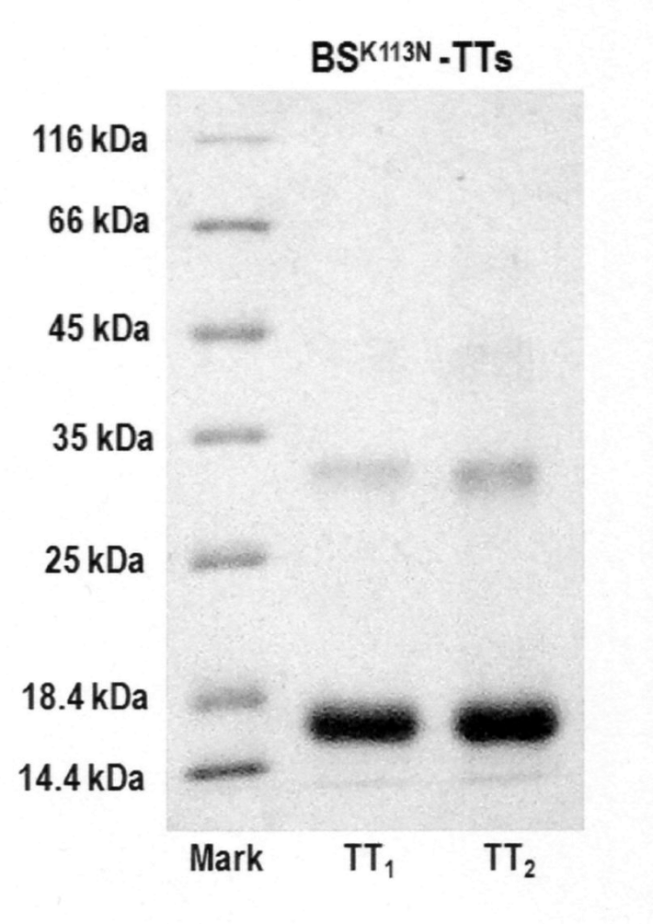

Supplement: Figure S3 — 10% acrylamide SDS-PAGE of BSK113N tetramers after their cross-linking with DFDNB. The lane corresponding to TT2 (considered totally N-swapped) shows a slightly higher amount of cross-linked products than the corresponding TT1. (TIF) [file pone.0046804.s003.tif]

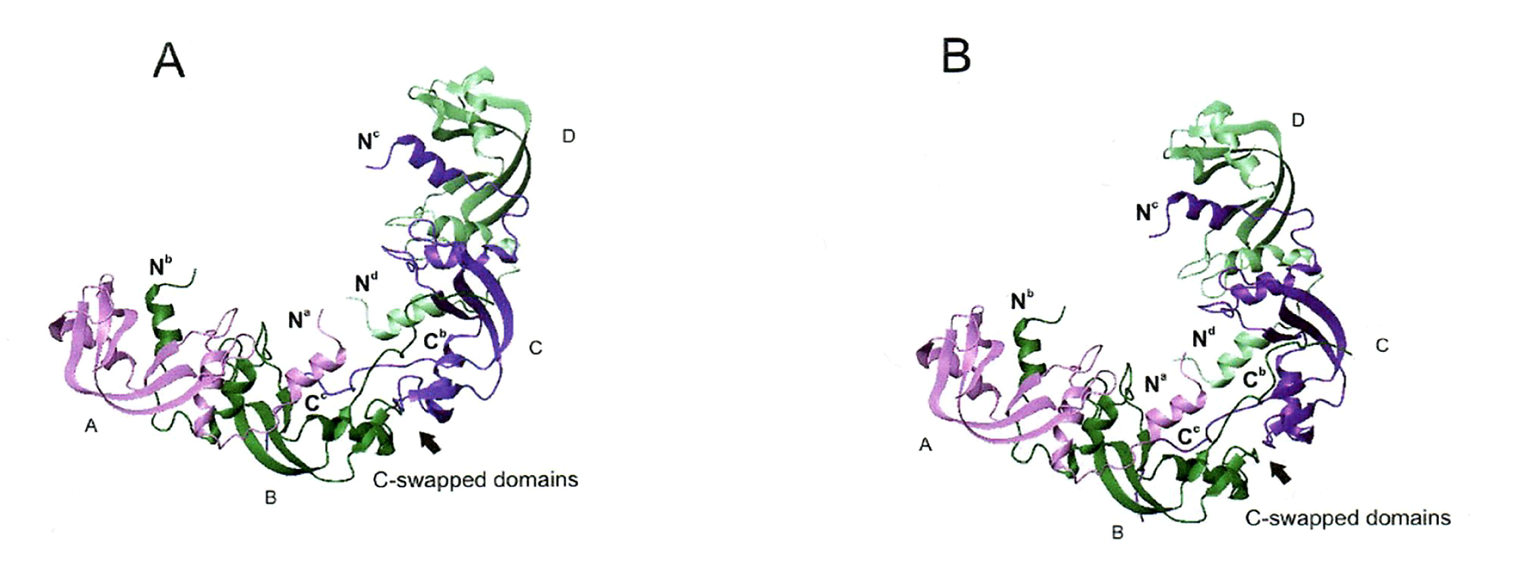

Supplement: Figure S4 — Alternative bent NCNTT models for TT1/C. The modeled structures (A,B) display an increasing central bending with respect to the one shown in Figure 6A, and represent energy minima as well as the latter, but their hydrodynamic diameter is less in agreement with the one experimentally measured for BS-TT1/C (Figure 1, Figure 5). (TIF) [file pone.0046804.s004.tif]

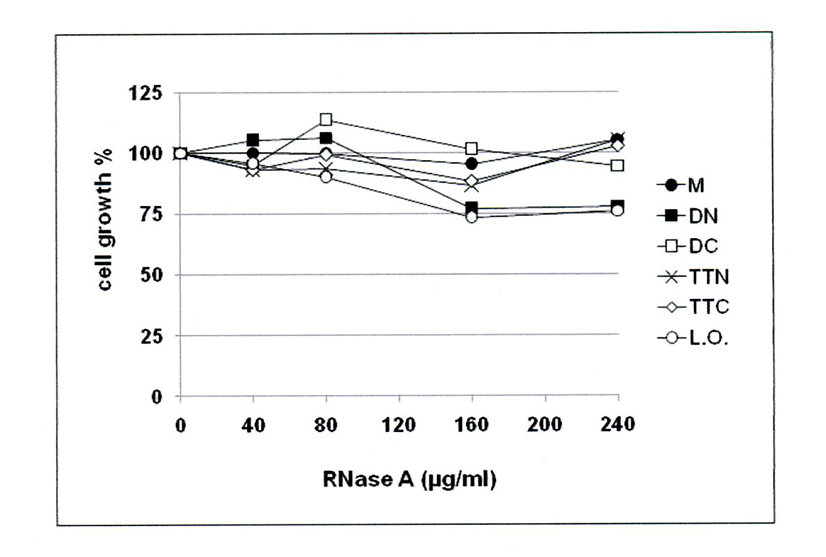

Supplement: Figure S5 — Action of RNase A oligomers on the proliferation of mesenchimal VIT1 cells. Cells were cultured in RPMI 1640 medium supplemented with 2 mM glutamine, 10% FBS, and 50 µg/ml gentamicin sulphate. After the RNase A species addition, 40 to 240 µg/ml, cells were incubated for 72 h at 37°C with 5% (v/v) CO2. At the end of the treatments cells were stained with a Crystal Violet solution and the survival was measured and compared to the control without any RNase species. RNase A species: M, monomer; DN, N-swapped dimer; DC, C-swapped dimer; TTN, NCN-swapped tetramer; TTC, CNC-swapped tetramer; L.O., mixture of RNase A pentamers, hexamers, and larger oligomers. (TIF) [file pone.0046804.s005.tif]
