# Supplementary material for: Double Domain Swapping in Bovine Seminal RNase: Formation of Distinct N- and C-swapped Tetramers and Multimers with Increasing Biological Activities
Source: PLoS One. 2012 Oct 11;7(10):e46804. doi: 10.1371/journal.pone.0046804 (PMC3469567; doi:10.1371/journal.pone.0046804)
Supplement: Discussion S1 — Discussion concerning the results derived from the patterns reported in Figure S2. (DOC) [file pone.0046804.s006.doc]

Supplementary Material For

**Double Domain** **Swapping in Bovine Seminal RNase: formation of distinct N- and C-swapped Tetramers and Multimers with Increasing Biological Activities**

Giovanni Gotte^*†^, Alexander Mahmoud Helmy^*^, Carmine Ercole^‡^, Roberta Spadaccini^§^, Douglas V. Laurents^||^, Massimo Donadelli*, Delia Picone^‡^

^*^Dipartimento di Scienze della Vita e della Riproduzione, Sezione di Chimica Biologica, Università degli Studi di Verona, Verona, Italy

^‡^ Dipartimento di Scienze Chimiche, Università degli Studi di Napoli “Federico II”, Naples, Italy

^§^ Dipartimento di Scienze Biologiche e Ambientali, Università del Sannio, Benevento, Italy.

^||^ Instituto de Química Física “Rocasolano” (C.S.I.C.), Madrid, Spain.

† E-mail: [giovanni.gotte@univr.it](mailto:giovanni.gotte@univr.it)

This file contains **Figure S2 discussion**

**Figure S2 discussion**

BS-RNase native dimer was cross-linked with DFDNB to verify if the N-term lock could furnish only C-swapped multimers. In particular, we used two different strategies: one with a slight excess of cross-linker and with 20 hours-long final stirring in the dark; unfortunately, most of the protein precipitated, while in a parallel experiment RNase A only slightly precipitated. Thus, a second attempt was performed with a DFDNB/protein ratio equal to the one suggested by the literature, but with only a 3h final stirring. In this case, only a slight precipitation occurred, and the clarified solution was purified allowing the isolation of four main fractions that were separately induced to oligomerize. The cross-linker should have not dramatically changed the protein Molecular Weight, so that the purification of the cross-linked mixture obtained with SEC suggested us to consider only the third fraction (“***3***” in Figure S2A, dotted line-pattern) out of the four main fractions visible (*1-4*). Anyhow, after inducing the self-association of this fraction, two tetramers were still present (panel A, continuous line-pattern), as occurs when starting from the native dimer (dashed line).

Consequently, the DFDNB-BS fraction ***3*** was further purified through cation-exchange chromatography [50], to separate the reacted protein from the un-reacted portion. In Figure S2B, continuous blue and red lines, are reported the patterns relative to two out of five conditions attempted (two with NaCl as eluent, three with NaPi): after this step, the DFDNB-BS-RNase purified, yellow fraction (panel B) was induced to oligomerize, following the procedure of Crestfield et al. [11], and the resulting sample was separated and analysed, again, with SEC.

The pattern (Figure S2C, continuous line) shows the presence of only one tetrameric peak, very probably TT_1_, suggesting that it could be the C-swapped one, as expected, and indicating that cation-exchange allowed us to better purify the cross-linked BS-RNase from the un-reacted one. Anyhow, the wideness of the tetrameric peak, and its position almost intermediate between BS-TT_1_ and TT_2_ (Figure S2C, dashed line), cannot allow us to consider it a clear-cut result. This could be even ascribable to the formation of several side-products induced by DFDNB cross-linking [49], but it has even to be considered that the modification of a RNase region directly involved in the domain swapping, as the N-terminus is, can affect the exchange of other domains too [53,62].
